# Identification of the functional variant driving *ORMDL3* and *GSDMB* expression in human chromosome 17q12-21 in primary biliary cholangitis

**DOI:** 10.1038/s41598-017-03067-3

**Published:** 2017-06-06

**Authors:** Yuki Hitomi, Kaname Kojima, Minae Kawashima, Yosuke Kawai, Nao Nishida, Yoshihiro Aiba, Michio Yasunami, Masao Nagasaki, Minoru Nakamura, Katsushi Tokunaga

**Affiliations:** 1 Department of Human Genetics, Graduate School of Medicine, the University of Tokyo, Tokyo, Japan; 2 Department of Integrative Genomics, Tohoku Medical Megabank Organization, Tohoku University, Sendai, Japan; 3 Graduate School of Medicine, Tohoku University, Sendai, Japan; 4 Japan Science and Technology Agency (JST), Kawaguchi, Japan; 5 The Research Center for Hepatitis and Immunology, National Center for Global Health and Medicine, Ichikawa, Japan; 6 Clinical Research Center, National Hospital Organization, Nagasaki Medical Center, Omura, Japan; 7 Department of Clinical Medicine, Institute of Tropical Medicine, Nagasaki University, Nagasaki, Japan; 8 Graduate School of Information Sciences, Tohoku University, Sendai, Japan; 9 Department of Hepatology, Nagasaki University Graduate School of Biomedical Sciences, Omura, Japan; 10 Headquarters of PBC Research in NHOSLJ, Clinical Research Center, National Hospital Organization Nagasaki Medical Center, Omura, Japan

## Abstract

Numerous genome-wide association studies (GWAS) have been performed to identify susceptibility genes to various human complex diseases. However, in many cases, neither a functional variant nor a disease susceptibility gene have been clarified. Here, we show an efficient approach for identification of a functional variant in a primary biliary cholangitis (PBC)-susceptible region, chromosome 17q12-21 (*ORMDL3*-*GSDMB*-*ZPBP2*-*IKZF3*). High-density association mapping was carried out based on SNP imputation analysis by using the whole-genome sequence data from a reference panel of 1,070 Japanese individuals (1KJPN), together with genotype data from our previous GWAS (PBC patients: n = 1,389; healthy controls: n = 1,508). Among 23 single nucleotide polymorphisms (SNPs) with *P* < 1.0 × 10^−8^, rs12946510 was identified as the functional variant that influences gene expression via alteration of Forkhead box protein O1 (FOXO1) binding affinity *in vitro*. Moreover, expression-quantitative trait locus (e-QTL) analyses showed that the PBC susceptibility allele of rs12946510 was significantly associated with lower endogenous expression of *ORMDL3* and *GSDMB* in whole blood and spleen. This study not only identified the functional variant in chr.17q12-21 and its molecular mechanism through which it conferred susceptibility to PBC, but it also illustrated an efficient systematic approach for post-GWAS analysis that is applicable to other complex diseases.

## Introduction

Up to the present time, thousands of genetic variants associated with complex human traits and diseases have been identified by genome-wide association study (GWAS)^1^. However, these variants often explain only a relatively small proportion of the heritability of the disease, which has led to the concept of “missing heritability”^2^. This missing heritability is considered to result from (i) limitations pertaining to detection power, (ii) sample availability, and (iii) other variants which cannot be detected by GWAS such as rare variants, small in/dels, and copy number variations. High-density association mapping based on single nucleotide polymorphism (SNP) imputation analysis has been able to explain some of the remaining missing heritability for human traits and disorders, e.g., for human height and body mass index^3^.

Primary biliary cholangitis (PBC) is a chronic and progressive cholestatic liver disease in which intrahepatic bile ducts are destroyed, accompanied by portal inflammation, liver cholangitis, and hepatic failure^4^. It is considered that these events are triggered by an autoimmune reaction against biliary epithelial cells. There is strong evidence to suggest that genetic factors contribute to PBC development. Thus, the estimated relative sibling risk (λs) in PBC is 10.5. Additionally, in identical twins, the concordance rate of PBC is higher than that in other autoimmune diseases^5, 6^. The genes associated with susceptibility to PBC have been identified as *Human Leukocyte Antigen (HLA)* and 31 non-*HLA* susceptibility regions (*IL12A*, *IL12RB2, STAT4, IRF5, MMEL1, SPIB, DENND1B, CD80, IL7R, CXCR5, TNFRSF1A, CLEC16A, NFKB1, RAD51L1, MAP3K7IP1, PLCL2, RPS6KA4, TNFAIP2, ELMO1, IRF8, TNFSF11, SH2B3, CRHR1, TYK2, IL1RL2, CCL20, DGKQ, C5orf30, LOC285626, TNFAIP3*, and chr.17q12-21) in GWAS and subsequent meta-analyses of subjects of European descent^7–14^.We recently identified *HLA*, *tumor necrosis factor superfamily member 15 (TNFSF15)*, *POU domain class 2 associating factor 1 (POU2AF1)*, *protein kinase C beta (PRKCB)*, *mannosidase beta A lysosomal (MANBA)*- *nuclear factor of kappa light polypeptide gene enhancer in B-cells 1 (NFKB1)*, *interleukin 7 receptor (IL7R)*, and the chr.17q12-21 locus in a GWAS for PBC in the Japanese population^15, 16^.

This 200-kb stretch of the genome is located in a strong linkage disequilibrium (LD) block structure in chr.17q12-21 (D’ > 0.9 and r^2^ > 0.6, See Supplementary Fig. 1), that includes *orosomucoid-like 3* (*ORMDL3*), *gasdermin B* (*GSDMB*), *zona pellucida binding protein 2* (*ZPBP2*), and *IKAROS family zinc finger 3* (*IKZF3*), and has been reported as one of the susceptibility gene regions that are associated with susceptibility to asthma, rheumatoid arthritis (RA), systemic lupus erythematosus (SLE), and inflammatory bowel disease (IBD) represented by rs2305480, rs2872507, rs1453560, and rs12946510, respectively, as well as PBC^17–22^. Among these genes, the function of *IKZF3* and *ORMDL3* gene products has been reported. *IKZF3* encodes a member of the Ikaros family of zinc-finger proteins, and functions as a transcription factor that is important for the regulation of B lymphocyte proliferation and differentiation^23^. The protein product of *ORMDL3* negatively regulates sphingolipid synthesis, regulates endoplasmic reticulum (ER)-mediated Ca^2+^ signaling, and is related to T-lymphocyte activation^24–26^. However, mainly because of the existence of an extended LD in this gene region (See Supplementary Fig. 1), it remains unclear what functional variants in the chr.17q12-21 locus underlie disease susceptibility to PBC and what the molecular mechanisms of such functional variants might be.

In the present study, in order to identify a functional variant in an extended LD block of human chromosome 17q12-21 in PBC, we performed a high-density association mapping based on SNP imputation analysis using a whole-genome sequence reference panel of 1,070 Japanese individuals and our previous GWAS data regarding PBC in the Japanese population. We next carried out a combination of *in silico* and *in vitro* functional analyses in this extended LD gene region to identify a functional variant. Finally, we performed expression-quantitative trait locus (e-QTL) analyses to clarify the molecular mechanisms underlying disease susceptibility to PBC via the functional variant.

## Results

### High-density association mapping based on SNP imputation analysis

**Figure 1:**
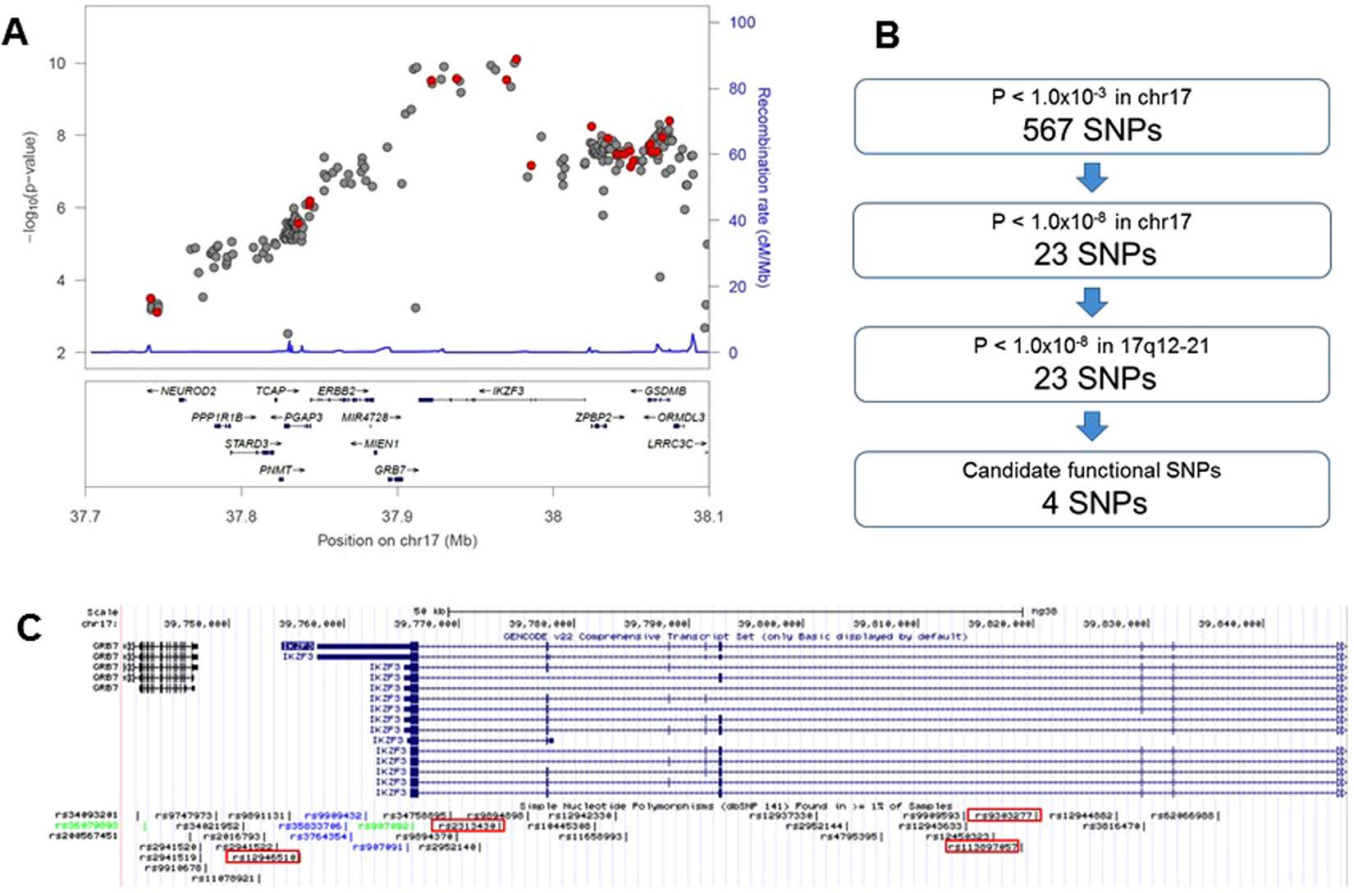
Selection of candidate functional variants in chr.17q12-21 that conferred susceptibility to PBC by using high-density association mapping. (**A**) Result of a high-density association mapping that was based on SNP imputation analysis using a whole-genome sequence reference panel of 1,070 Japanese individuals from a prospective, general population cohort study (1KJPN) and genotype data from our previous GWAS (PBC patients: n = 1,389; healthy controls: n = 1,508). The p-value of each SNP is indicated by a dot. Red dots show experimentally genotyped SNPs by GWAS. (**B**) Scheme of SNP selection based on *in silico* functional analysis. There were 567 and 23 SNPs whose P values were less than 1.0 × 10^−3^ and 1.0 × 10^−8^, respectively, that were located in chromosome 17. The latter 23 SNPs in chromosome 17 are all located in chr.17q12-21. Of these 23 SNPs, 2 SNPs (rs9303277 and rs113897057), which showed the most significant associations with susceptibility to PBC, and the candidate primary functional variants (rs2313430 and rs12946510) were chosen. These SNPs are located in a transcription regulatory element as assessed by the presence of a DNase I hyper-sensitivity cluster or H3K27Ac marks, and/or their location in a region that might modulate transcription factor binding. (**C**) Positions of the four candidate functional variants in the chr.17q12-21 locus. Candidates are shown in the red rectangles.

To perform high-density association mapping for the disease susceptibility region chr.17q12-21 to PBC in the Japanese population, SNP imputation analysis was performed using a reference panel of 1,070 Japanese individuals (1KJPN; Tohoku Medical Megabank Organization (ToMMo), Tohoku University, Japan) and genotype data from our previous GWAS (See Supplementary Fig. 2)^16, 27^. As shown in Table 1 and Fig. 1A and B, besides the SNPs that showed genome-wide significant associations in our previous GWAS (rs9303277, rs10445308, rs9909593, rs907091, and rs11557466)^15^, an additional 18 SNPs (rs113897057, rs4795395, rs2952144, rs2313430, rs12946510, rs2952140, rs12942330, rs2941522, rs12450323, rs907092, rs11658993, rs11078921, rs9747973, rs7219923, rs12150079, rs2872516, rs869402, and rs9303279) showed p-values that were less than 1.0 × 10^−8^ by SNP imputation analysis. Among these SNPs, four SNPs were located in *GSDMB*, two in *ZPBP2*, two in the intergenic region between *IKZF3* and *growth factor receptor bound protein 7* (*GRB7*), which is located near the strong LD block structure in chr.17q12-21, and the others were located in *IKZF3*.

**Table 1:** High-density association mapping of SNPs located in chr.17q12-21 that are associated with susceptibility to PBC in the Japanese population.

SNP_ID^a^	GWAS or Imputation^b^	Position (Chr.17)^c^	P^d^	OR^e^	Regulome DB^f^	UCSC^g^	Location
**rs9303277**	GWAS	37976469	7.66E-11	1.434	1f	×	*IKZF3* Intron 3
**rs113897057**	Imputation	37975214	1.00E-10	1.431	5	○	*IKZF3* Intron 3
rs4795395	Imputation	37962987	1.04E-10	1.432	—	×	*IKZF3* Intron 3
rs2952144	Imputation	37960017	1.09E-10	1.432	6	×	*IKZF3* Intron 3
**rs2313430**	Imputation	37929816	1.78E-10	1.426	1d	○	*IKZF3* Intron 7
**rs12946510**	Imputation	37912377	1.94E-10	1.427	1b	◎	Intergenic (*IKZF3/GRB7*)
rs2952140	Imputation	37928059	1.95E-10	1.426	5	×	*IKZF3* Intron 7
rs12942330	Imputation	37939839	2.07E-10	1.427	6	×	*IKZF3* Intron 6
rs10445308	GWAS	37938047	2.67E-10	1.421	1f	×	*IKZF3* Intron 6
rs2941522	Imputation	37910368	2.71E-10	1.421	4	○	Intergenic (*IKZF3/GRB7*)
rs9909593	GWAS	37970149	2.93E-10	1.419	1f	×	*IKZF3* Intron 3
rs907091	GWAS	37921742	3.07E-10	1.419	—	−	*IKZF3* 3′UTR
rs12450323	Imputation	37972708	3.61E-10	1.409	5	×	*IKZF3* Intron 3
rs907092	Imputation	37922259	5.24E-10	1.417	—	−	*IKZF3* Exon 8 (synonymous)
rs11658993	Imputation	37940808	1.12E-09	1.409	—	×	*IKZF3* Intron 6
rs11078921	Imputation	37908867	2.14E-09	1.459	5	×	Intergenic (*IKZF3/GRB7*)
rs9747973	Imputation	37905107	2.24E-09	1.396	5	△	Intergenic (*IKZF3/GRB7*)
rs7219923	Imputation	38074518	4.02E-09	1.409	1f	△	*GSDMB* Intron 1
rs11557466	GWAS	38024626	5.58E-09	1.409	—	−	*ZPBP2* Exon 2 (synonymous)
rs12150079	Imputation	38025417	8.15E-09	1.465	1f	×	*ZPBP2* intron 2
rs2872516	Imputation	38072727	8.28E-09	1.402	5	×	*GSDMB* Intron 2
rs869402	Imputation	38068043	8.52E-09	1.401	6	×	*GSDMB* Intron 3
rs9303279	Imputation	38073968	9.78E-09	1.399	6	△	*GSDMB* Intron 1

^a^SNPs shown in bold were the final candidate for primary functional variants.

^b^Genotyped by our previous GWAS (ref. 16) or the imputed genotypes by the high-density association mapping in the present study.

^c^Position of the SNPs in hg19.

^d^P values calculated for the allelic model using Pearson’s Chi-square test.

^e^Odds ratio (OR) of the minor allele as calculated from the two-by-two allele frequency table.

^f^Functinoal prediction scores of each SNPs by RegulomeDB database.

^g^Probability of the functional damages checked by UCSC genome browser.

### *In silico* functional analysis to select candidates for functional variants

From the total of 23 SNPs, SNPs that were predicted to be located in transcription regulatory elements, which were characterized by DNase hyper-sensitivity cluster analyses, SNPs that were predicted to be binding sites of transcription factors, and SNPs that showed significant associations in e-QTL analysis, were selected using the Regulome DB database (http://www.regulomedb.org/index)^28^. Next, seven SNPs with Regulome DB scores higher than 2a (supported by the data of e-QTL and transcription factor binding/DNase peak) were selected as potential candidates (Table 1; rs9303277 and rs9909593 in intron 3 of *IKZF3*, rs10445308 in intron 6 of *IKZF3*, rs2313430 in intron 7 of *IKZF3*, rs7219923 in intron 1 of *GSDMB*, rs12150079 in intron 2 of *ZPBP2*, and rs12946510 in the intergenic region between *GRB7* and *IKZF3*). Among these seven SNPs, two SNPs (Table 1, rs2313430 and rs12946510) that were located in transcription regulatory elements defined by (1) DNase I hyper-sensitivity cluster analysis of any one of 125 cell types and (2) by the existence of H3K27Ac markers in any one of the cell lines (GM12878, H1-hESC, HSMM, HUVEC, K562, NHEK, or NHLF) were selected as the final candidates using the UCSC genome browser (http://genome.ucsc.edu/index.html)^29^. In addition to these two SNPs, the two most significant SNPs in the high-density association mapping (Table 1, rs9303277 and rs113897057), were also selected as final-candidate primary variants in the chr.17q12-21 locus for the conferral of susceptibility to PBC (Fig. 1B and C).

### *In vitro* functional analysis to identify the functional variant

**Figure 2:**
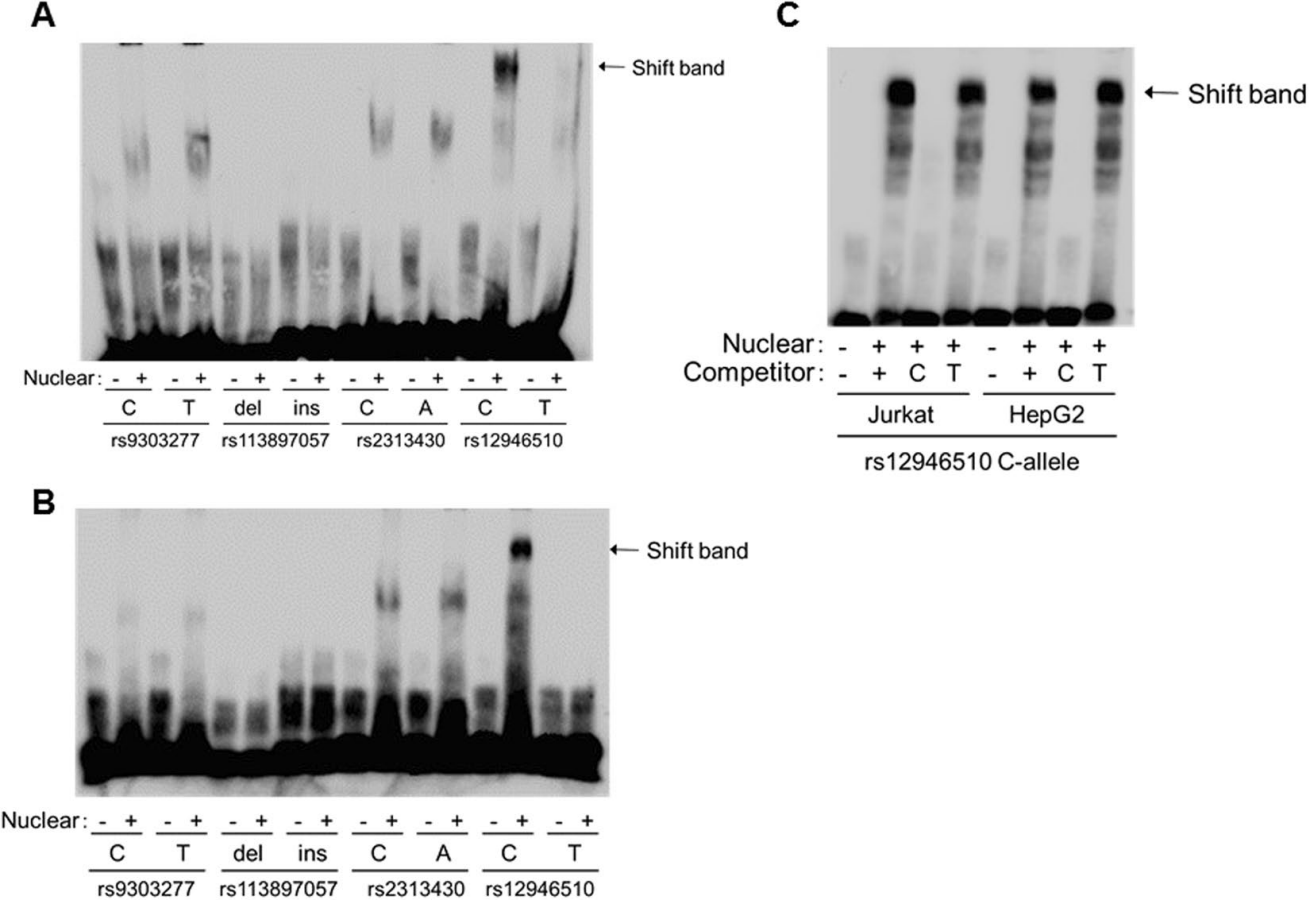
Analysis of each candidate functional variant using electrophoretic mobility shift assays (EMSA). (**A** and **B**) EMSA of each of the four candidate primary variants using biotin-labeled probes corresponding to the major alleles and the minor alleles, and nuclear extracts of Jurkat (human T lymphocyte) (**A**) and HepG2 (human liver carcinoma) (**B**) cells. Rs12946510 was the only variant to show a difference in mobility shift between the major allele (C-allele) and the PBC susceptibility allele (T-allele). (**C**) Competitor assay using a 200× amount of unlabeled probe corresponding to the major or the minor alleles. Three independent experiments were performed in each assay.

**Figure 3:**
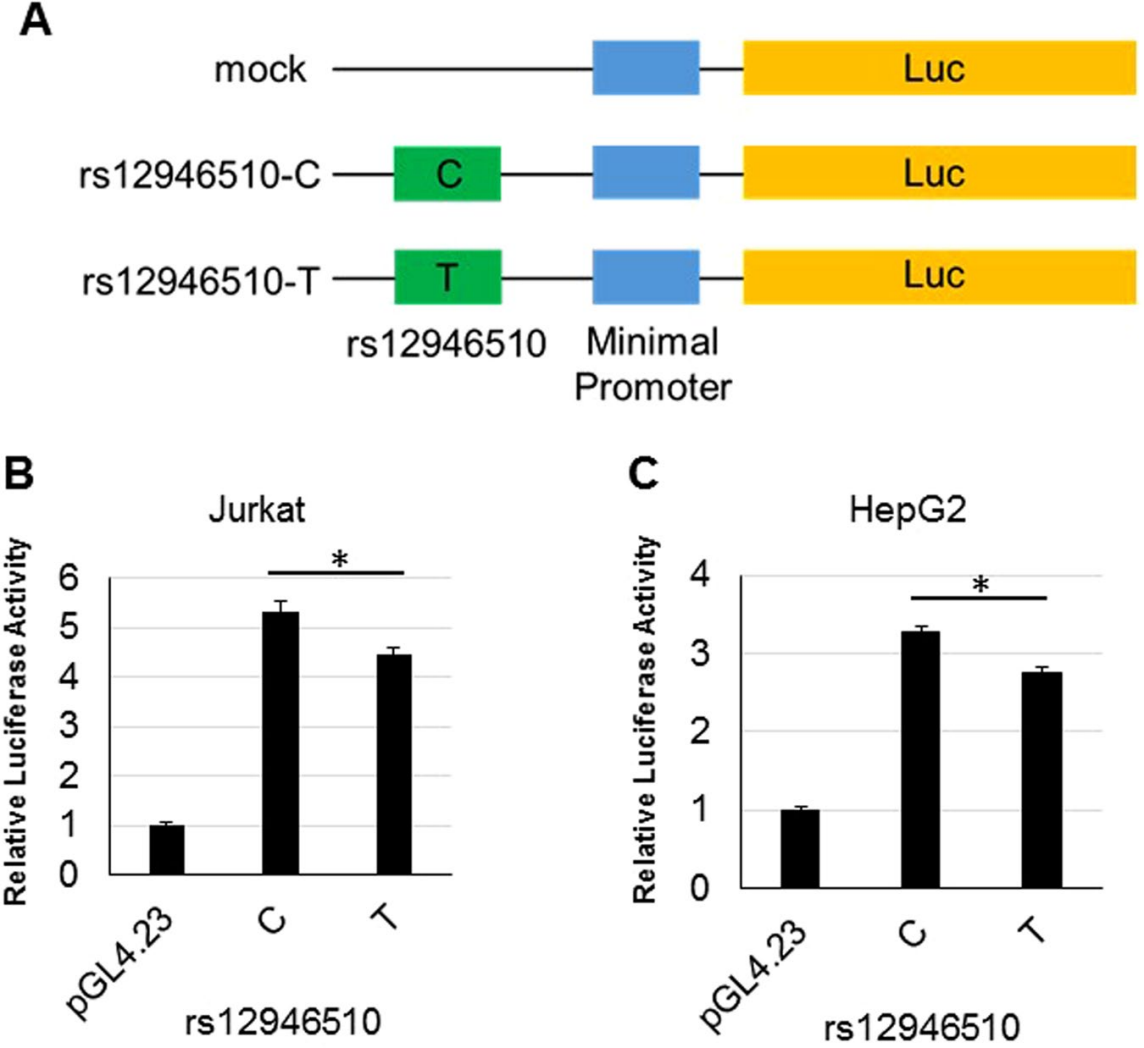
Luciferase reporter assay of rs12946510. (**A**) Outline of the luciferase reporter plasmid constructs used for transfection. The plasmids consisted of PCR fragments of the genomic DNA between *GRB7* and *IKZF3* containing rs12946510 that were sub-cloned into the pGL4.23 vector. (**B** and **C**) The ability of these plasmid constructs to enhance transcription in transfected Jurkat (**B**) and HepG2 (**C**) cells was measured by determination of cellular luciferase (luc) activity 24 hours after transfection. The luciferase activities of cells transfected with the PBC susceptibility allele (T-allele) of rs12946510 were reduced compared to those transfected with the major allele (C-allele). Three independent experiments were performed in each assay. The data in the figures represent averages ± standard deviation of triplicate assays in one experiment. *P < 0.001 (Student’s t test).

To evaluate the effect of the final-candidate primary variants on binding affinities of transcription factors, biotin-labeled probes corresponding to the different alleles of each SNP were used in an electrophoretic mobility shift assay (EMSA) with nuclear extracts of the Jurkat (human T lymphocyte) and HepG2 (human liver carcinoma) cell lines. This assay detected a difference in mobility shift between the major and the minor (i.e., PBC-susceptible) alleles of rs12946510. No difference in mobility shift was detected for the other alleles tested (rs9303277, rs113897057, and rs2313430) (Fig. 2A and B). The band shifted by the major allele (i.e., C-allele) of rs12946510 was specifically diminished by incubation with a 200× amount of a non-labeled competitor probe (Fig. 2C). Luciferase reporter assays were used to further determine differences in transcription efficiency between the major and the PBC susceptibility alleles of rs12946510. These assays were performed in the Jurkat, HepG2, and the human bile duct carcinoma, HuCCT1, cell lines (Fig. 3A–C, and See Supplementary Fig. 3). In all cell lines, the luciferase activity of cells transfected with the reporter construct containing the PBC susceptibility allele of rs12946510 was significantly reduced to 70% of that of cells transfected with the reporter construct containing the major allele at 24 hours after transfection of the pGL4.23 vector. Similar results were obtained in the luciferase assay when a construct was used in which the inserted sequence containing rs12946510 was inserted in an opposite orientation (see Supplementary Fig. 4). These results indicated that rs12946510, which is located in the intergenic region between *GRB7* and *IKZF3*, was the functional variant in the enhancer region in chr.17q12-21 which conferred susceptibility to PBC.

### Molecular mechanisms underlying the PBC susceptibility allele of rs12946510

**Figure 4:**
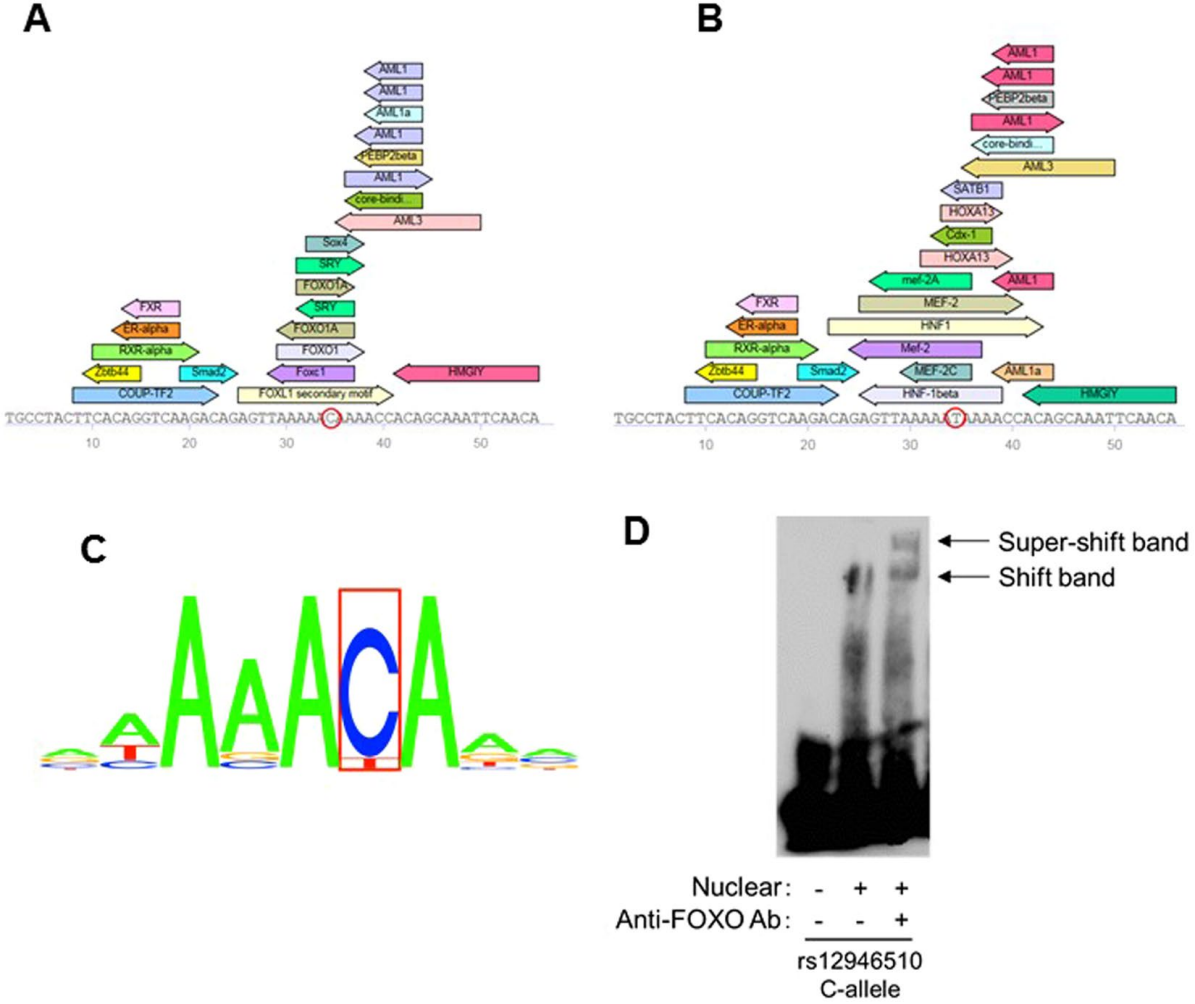
Analysis of FOXO1 binding using a super-shift assay. (**A** and **B**) Prediction of transcription factor binding by the TRANSFAC professional database. Although the sequence of the FOXO1 binding site contains the major allele of rs12946510 (C allele, **A**), this sequence was disrupted by the PBC- susceptibility allele (T allele, (**B**). (**C**) The binding motif of FOXO1. The nucleotide that is shown inside the rectangle is the position of rs12946510. (**D**) Incubation of the Jurkat cell nuclear extract with a specific anti-FOXO1 antibody resulted in a super-shift of the shifted band that was observed in the EMSA. Three independent experiments were performed in each assay.

Based on analysis using the TRANSFAC professional database it was predicted that the major allele of rs12946510 but not the PBC susceptibility allele constituted a Forkhead box protein O1 (FOXO1) binding motif (Fig. 4A–C, http://www.gene-regulation.com/pub/databases.html)^30^. In addition, the same alteration of FOXO1 binding between rs12946510 major and minor alleles was predicted by RegulomeDB. Therefore, the binding of FOXO1 with the major allele of rs12946510 was investigated in a super-shift assay using the nuclear extract of Jurkat. In accordance with the results of the predictions of transcription factor binding as described above, the shifted band caused by the major allele of rs12946510 was super-shifted by pre-incubation with an anti-FOXO1 specific antibody before electrophoresis (Fig. 4D). This result suggested that, in the PBC susceptibility allele of rs12946510, a primary FOXO1 binding site was disrupted. Thus, rs12946510 could function as the functional variant that regulates the efficiency of the FOXO1-related gene expression enhancer.

**Figure 5:**
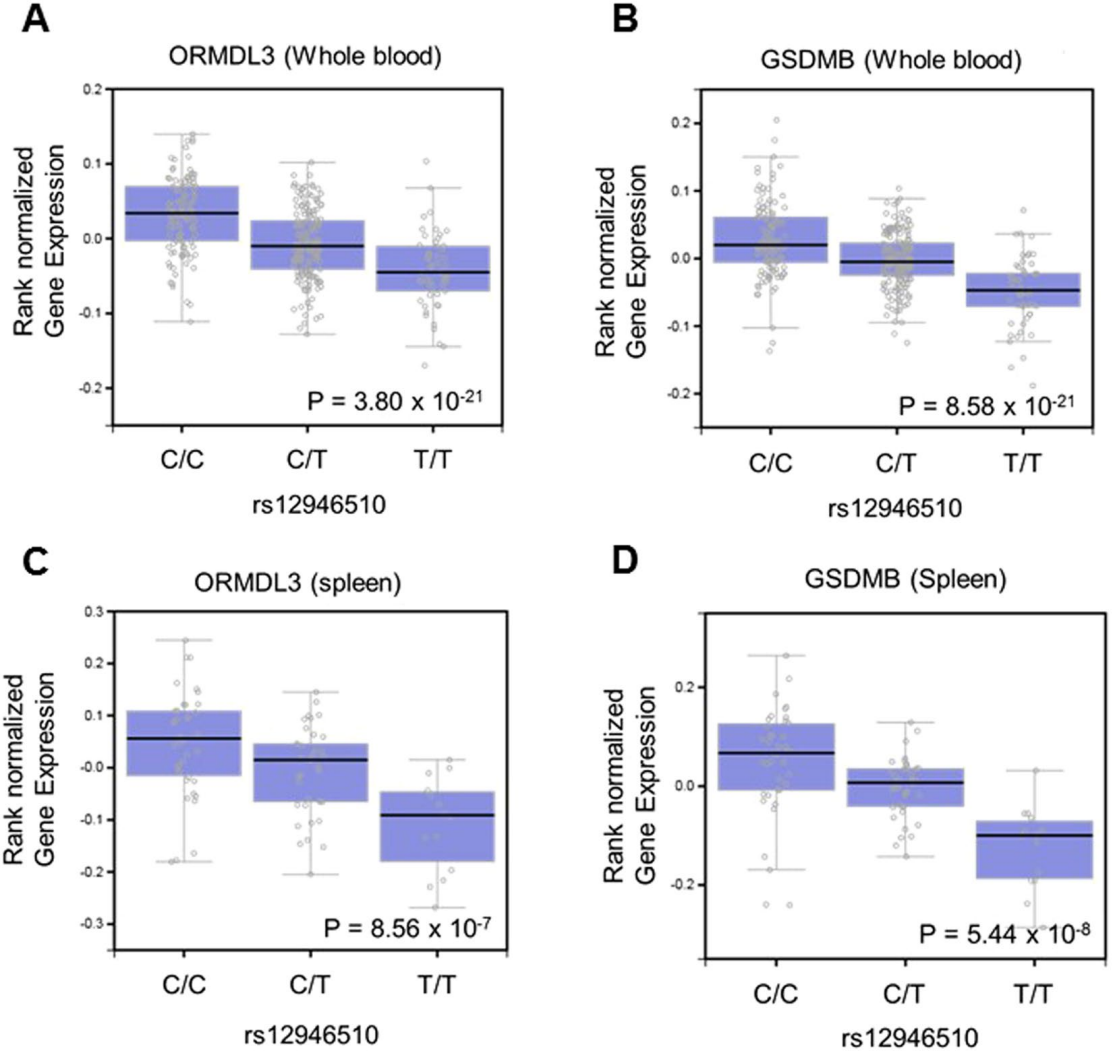
rs12946510 genotypes are associated with differences in endogenous *ORMDL3* and *GSDMB* expression levels. (**A** and **B**) Endogenous expression level of *ORMDL3* (**A**) and *GSDMB* (**B**) in whole-blood. (**C** and **D**) Endogenous expression level of *ORMDL3* (**C**) and *GSDMB* (**D**) in the spleen. Individuals with the rs12946510 susceptibility allele showed reduced endogenous *ORMDL3* and *GSDMB* expression levels compared to individuals with the major allele. The statistical significance level after multiple comparison compensation by Bonferroni correction was *P* = 0.0045. These data were extracted from the GTEx portal database.

To assess the influences of rs12946510 on gene expression efficiency, the endogenous expression levels of all of the genes located in chromosome 17 were compared in whole blood and spleen among the genotypes of rs12946510 using the GTEx portal database (http://gtexportal.org/home/)^31^. Individuals with PBC-susceptible genotypes (i.e. CT and TT) of rs12946510 showed significant decreases in the expression level of *ORMDL3* and *GSDMB* in whole blood (Fig. 5A and B; n = 338; *P* = 3.80 × 10^−21^ and *P* = 8.58 × 10^−21^, respectively) and in the spleen (Fig. 5C and D; n = 89; *P* = 8.56 × 10^−7^ and 5.44 × 10^−8^, respectively). The p-values calculated for these genes were the lowest among the p-values of all of the genes located in chromosome 17. However, individuals with the PBC-susceptible genotypes of rs12946510 did not show any significant decrease or increase in the expression levels of *IKZF3* in whole blood (See Supplementary Fig. 4A) or spleen (See Supplementary Fig. 5A). Although the data of *ZPBP2* in whole blood are not available, *ZPBP2* expression levels did not show a significance difference among the genotypes of rs12946510 in the spleen (See Supplementary Fig. 5B). The expression levels of *GRB7* and *gasdermin A* (*GSDMA*), which are located outside but close to the strong LD block structure in chr.17q12-21 did not show significance differences among the genotypes of rs12946510 in whole blood (See Supplementary Fig. 4B and C) or spleen (See Supplementary Fig. 5C and D).

Additionally, chromatin interaction between the 5 kb window that contains rs12946510 and the upstream sequence of *ORMDL3* and *GSDMB* could be detected in Hi-C database analysis (http://promoter.bx.psu.edu/hi-c/index.html) (see Supplementary Fig. 7)^32^.

These results suggested that in individuals with the PBC‑susceptible genotype of rs12946510, organs related with the immune response had reduced endogenous *ORMDL3* and *GSDMB* expression levels.

## Discussion

**Figure 6:**
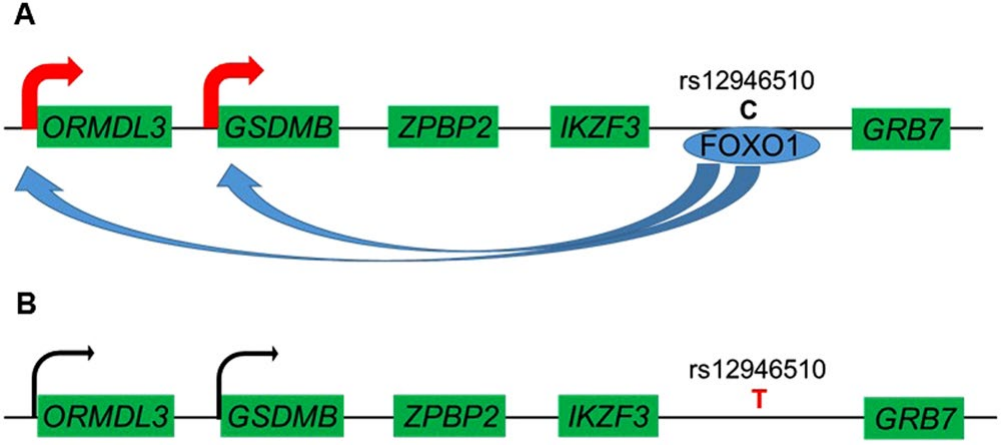
The potential functional effect of the major (C-allele) and PBC susceptibility allele (T-allele) of rs12946510. (**A**) The FOXO1 binding motif is located around the major allele (C-allele) of rs12946510 in the intergenic region between *GRB7* and *IKZF3*, and probably enhances the expression of *GSDMB* and *ORMDL3*. (**B**) In the PBC susceptibility rs12946510-T allele the binding site for FOXO1 is disrupted.

The current study, which included high-density association mapping based on SNP imputation analysis and *in silico/in vitro* functional analyses, identified rs12946510 as the functional variant in chr.17q12-21, a well-known PBC-susceptibility region in multiple ethnicities^8, 12–14, 16^. Super-shift assay analysis demonstrated that the disease susceptibility allele of rs12946510 disrupted the primary FOXO1 binding motif. Additionally, the whole blood and spleen from individuals with the PBC susceptibility allele of rs12946510 showed significantly lower *ORMDL3* and *GSDMB* expression levels than individuals without this allele. These results showed that the PBC susceptibility allele of rs12946510 disrupted the enhancer region for *ORMDL3* and *GSDMB* gene expression (Fig. 6). Chromatin interaction between the sequence that contains rs12946510 and the upstream sequence of *ORMDL3* and *GSDMB* also supported this enhancer model (see Supplementary Fig. 7)^32^.

Recently, emerging methods of SNP imputation using a large scale reference panel have been used as an efficient approach for supplementation of GWAS data to identify disease-associated genetic factors^33^. In order to improve the accuracy of the results of SNP imputation analysis, large sample sizes are needed for both the GWAS and the whole-genome sequence reference panels^34^. Since the whole-genome sequencing of 104 JPT (Japanese living in Tokyo) by the 1000 Genome project (http://www.1000genomes.org/) showed that the proportion of the variants that were characteristic to the Japanese population was higher than that in other populations, even in East Asia^35^, it became clear that ethnicity-matched reference data of whole-genome sequences obtained from a large number of individuals was needed for appropriate SNP imputation analysis. We recently conducted an analysis of the whole-genome sequences of 1,070 Japanese individuals using high-coverage next-generation sequencing (32.4x on average) and constructed the largest Japanese population reference panel (1KJPN)^27^. By using this 1KJPN we were able to identify functional variants by high-density association mapping^16^.

Unlike gene expression promoter regions that are located upstream of the transcription start site, expression enhancer regions are located not only within or near the genes whose expression is regulated, but also at a distance of more than 100 kb from the regulated genes, as exemplified by the enhancer regions of mouse *sox2* ^36^. In the present study, we clearly showed that, although rs12946510 was located between *IKZF3* and *GRB7*, it affects the expression levels of other genes such as *ORMDL3* and *GSDMB* that are located 120 kb downstream from the SNP. Thus, our approach consisting of high-density association mapping and *in silico/in vitro* analyses could overcome the difficulties of genetic analysis that are caused by the strong LD block structure.

*ORMDL3* has been reported as one of the susceptibility genes to asthma (the most significantly associated SNP of *ORMDL3* was rs7216389)^17, 18^. Individuals with the asthma- susceptibility allele of rs7216389 showed a higher *ORMDL3* expression level than those with an asthma-protective allele^17^. In contrast, the asthma- susceptibility allele of rs2872507 (located in chr.17q12-21, r^2^ = 0.96 with rs7216389 in 1000 genomes panels in Asian subjects) was protective against autoimmune diseases such as rheumatoid arthritis, Crohn’s disease, and ulcerative colitis^37^. In addition, other SNPs such as rs8076131 and rs4065275, which are located in *ORMDL3* and are associated with the endogenous expression levels of *ORMDL3*, were recently reported to affect the levels of type-2 helper T cell (Th2) cytokines such as interleukin-4 (IL-4) and interleukin-13 (IL-13) in the supernatants of stimulated PBMCs^38^. This evidence indicated that the difference in *ORMDL3* expression levels is directly related to the balance of Th subsets and Th cytokine levels and eventually confers disease susceptibility to asthma and other autoimmune diseases. In the present study, in accordance with these previous reports, the PBC- susceptibility allele of rs12946510 showed a lower expression level of *ORMDL3*, which is possibly followed by lower expression levels of Th2 subset/cytokines and the predominance of other subsets/cytokines of Th *in vivo*, which are closely associated with the pathogenesis of PBC^39^. These observations indicated that *ORMDL3* potentially has important protective effects against the development of PBC. Further evaluation of the expression levels of *ORMDL3* in immunological organs derived from PBC patients will confirm our present hypothesis.

In conclusion, the *in silico/in vitro* functional analyses in the present study showed the molecular mechanisms through which rs12946510 caused reductions in the transcriptional efficiency of *ORMDL3* and *GSDMB*, i.e. the transcription factor FOXO1 showed a possible protective effect against the development of PBC by binding to the expression enhancer region which includes rs12946510. These results indicated that *ORMDL3*, *GSDMB*, and *FOXO1* have potentially important roles for protection against the development of PBC. This evidence suggested that the functional changes resulting from primary variants could be clarified by focusing on the functional effect of primary functional variants and that such an approach could be decisive for understanding the molecular mechanisms by which diseases develop. Other than the gene locus that was the focus of the present study, *HLA*, *TNFSF15*, *POU2AF1*, *NFKB1-MANBA*, *PRKCB*, and *IL7R* also displayed significant associations with susceptibility to PBC in the Japanese population^16^. Among these genes, we already reported the functional variants of *TNFSF15* and *PRKCB* and their underlying molecular mechanisms^16, 40^. A similar systematic approach using the methods exemplified in the present study and focusing on the other and/or the as yet un-identified susceptibility genes to PBC is needed to clarify the molecular mechanisms of disease development.

## Materials and Methods

### Subjects, guidelines, and regulations

Information regarding the study participants, (PBC patients and healthy controls), was provided in a previous study^16^. Written informed consent for participation in this study was obtained from all participants. This study was approved by the Research Ethics Committee, and the committee on genetically modified organisms, of the Graduate School of Medicine, The University of Tokyo. All methods were performed in accordance with the ethical guidelines and regulations.

### Genotype imputation

SNP genotypes of PBC cases and healthy controls that passed SNP filtering were phased with SHAPEIT2 (v2.r644)^41^. The following options were used for SHAPEIT2: –burn 10, –prune 10, and –main 25. Genotype imputation was performed on the phased genotypes with IMPUTE2 (ver. 2.3.1)^42^ using a phased reference panel of 1,070 Japanese individuals from a prospective, general population^27^. For IMPUTE2, the following options were used: -Ne 2000, -k_hap 1000, -k 120, -burnin 15, and -iter 50.

### *In silico* functional analysis

The probability that candidate functional variants might influence transcription regulation was evaluated by using the RegulomeDB database (http://www.regulomedb.org/index)^28^ and the UCSC genome browser (http://genome.ucsc.edu/index.html)^29^. Transcription factor binding was predicted by TRANSFAC Professional (QIAGEN, Valencia CA; http://www.gene-regulation.com/pub/databases.html)^30^.

### Electrophoretic mobility shift assay (EMSA)

The LightShift Chemiluminescent EMSA Kit (Thermo-Fisher Scientific, Waltham, MA) was used to perform EMSA as per the manufacturer’s instructions. Biotin-labeled double-stranded oligonucleotide probes corresponding to each major and minor allele, whose sequences are provided in Supplementary Table 1, were used for this assay. These biotin-labeled probes (10 fmol/μl) were incubated with a nuclear extract (2.5 μg/ml) of Jurkat or HepG2 cells (Nuclear Extract Kit; Active Region, Carlsbad, CA) for 30 min at 25 °C.

The super-shift experiments were performed by incubating goat anti-human FOXO1 (FKHR) antibody (2 μg/μl; C-20 X; Santa Cruz Biotechnologies, Santa Cruz, CA) for 30 min at 25 °C with the mixture of the nuclear extract incubated with the biotin-labeled probe. Each assay was performed independently, three times.

### Luciferase reporter assay

For the luciferase assays, specific PCR primers (see Supplementary Table 2) were used to amplify part of the intergenic region between *IKZF3* and *GRB7* from human genomic DNA, which was then subcloned into the luciferase reporter pGL4.23 (luc2/minP) vector (Promega, Madison, Wis). pGL4.23 (500 ng) and the pGL4.74 (hRluc/TK) vector (50 ng) that was used as an internal control to account for cell numbers were transfected into Jurkat, HepG2, or HuCCT1 cells using Lipofectamine 3000 reagents (Thermo-Fisher Scientific). The Dual-Luciferase Reporter Assay system (Promega, Madison, WI) was used to measure luciferase activities. Each figure shows representative data of experiments that were done independently three times. The data in the figures represent averages ± standard deviation of triplicate assays in one experiment.

### eQTL

The correlation between the rs12946510 genotype and expression of all of the genes in chromosome 17 was examined using data available from the database of the GTEx portal at the BROAD Institute (http://gtexportal.org/home/)^31^.

### Hi-C

Chromatin interaction between the 5 kb window that contains rs12946510 and the upstream sequence of *ORMDL3* and *GSDMB* was examined using data available from the Hi-C database (http://promoter.bx.psu.edu/hi-c/index.html) at the ENCODE Consortium^32^.

### Statistical analysis

Statistical significance of the difference in luciferase activity between major and PBC susceptibility alleles of rs12946510 was determined using Student’s t-test. Multiple comparisons in e-QTL analysis were adjusted by the Bonferroni correction.

## Electronic supplementary material

Supplementary data

## References

[1] Welter D (2014). The NHGRI GWAS Catalog, a curated resource of SNP-trait associations. Nucleic Acids Res..

[2] Manolio TA (2009). Finding the missing heritability of complex diseases. Nature.

[3] Yang J (2015). Genetic variance estimation with imputed variants finds negligible missing heritability for human height and body mass index. Nat. Genet..

[4] Kaplan MM, Gershwin ME (2005). Primary biliary cholangitis. N. Engl. J. Med..

[5] Jones DE, Watt FE, Metcalf JV, Bassendine MF, James OF (1999). Familial primary biliary cholangitis reassessed: a geographicallybased population study. J. Hepatol..

[6] Selmi C (2004). Primary biliary cholangitis in monozygotic and dizygotic twins: genetics, epigenetics, and environment. Gastroenterology.

[7] Hirschfield GM (2009). Primary biliary cholangitis associated with HLA, IL12A, and IL12RB2 variants. N. Engl. J. Med..

[8] Hirschfield GM (2010). Variants at IRF5-TNPO3, 17q12-21 and MMEL1 are associated with primary biliary cholangitis. Nat. Genet..

[9] Liu X (2010). Genome-wide meta-analyses identify three loci associated with primary biliary cholangitis. Nat. Genet..

[10] Mells GF (2011). Genome-wide association study identifies 12 new susceptibility loci for primary biliary cholangitis. Nat. Genet..

[11] Hirschfield GM (2012). Association of primary biliary cholangitis with variants in the CLEC16A, SOCS1, SPIB and SIAE immunomodulatory genes. Genes Immun..

[12] Liu JZ (2012). Dense fine-mapping study identifies new susceptibility loci for primary biliary cholangitis. Nat. Genet..

[13] Juran BD (2012). Immunochip analyses identify a novel risk locus for primary biliary cirryosis at 13q14, multiple independent associations at four established risk loci and epistasis between 1p31 and 7q32 risk variants. Hum. Mol. Genet..

[14] Cordell HJ (2015). International genome-wide meta-analysis identifies new primary biliary cholangitis risk loci and targetable pathogenic pathways. Nat. Commun..

[15] Nakamura M (2012). Genome-wide association study identified TNFSF15 and POU2AF1 as susceptibility locus for primary biliary cholangitis in the Japanese population. Am. J. Hum. Genet..

[16] Kawashima, M. *et al*. Genome-wide association study identified PRKCB as a genetic susceptibility locus for primary biliary cholangitis in a Japanese population. *Hum. Mol. Genet*. **26**, 650–659 (2017). 10.1093/hmg/ddw406 28062665

[17] Moffatt MF (2007). Genetic variants regulating ORMDL3 expression contribute to the risk of childhood asthma. Nature..

[18] Moffatt MF (2010). A large-scale, consortium-based genomewide association study of asthma. N. Engl. J. Med..

[19] Kurreeman FA (2012). Use of a multiethnic approach to identify rheumatoid- arthritis-susceptibility locus, 1p36 and 17q12. Am. J. Hum. Genet..

[20] Lessard CJ (2012). Identification of IRF8, TMEM39A, and IKZF3-ZPBP2 as susceptibility locus for systemic lupus erythematosus in a large-scale multiracial replication study. Am. J. Hum. Genet..

[21] Barrett JC (2008). Genome-wide association defines more than 30 distinct susceptibility locus for Crohn’s disease. Nat. Genet..

[22] McGovern DP (2010). Genome-wide association identifies multiple ulcerative colitis susceptibility locus. Nat. Genet..

[23] Cortes M, Georgopoulos K (2004). Aiolos is required for the generation of high affinity bone marrow plasma cells responsible for long-term immunity. J. Exp. Med..

[24] Breslow DK (2010). Orm family proteins mediate sphingolipid homeostasis. Nature.

[25] Cantero-Recasens G, Fandos C, Rubio-Moscardo F, Valverde MA, Vicente R (2010). The asthma-associated ORMDL3 gene product regulates endoplasmic reticulum-mediated calcium signaling and cellular stress. Hum. Mol. Genet..

[26] Carreras-Sureda A (2013). ORMDL3 modulates store-operated calcium entry and lymphocyte activation. Hum. Mol. Genet..

[27] Nagasaki M (2015). Rare variant discovery by deep whole-genome sequencing of 1,070 Japanese individuals. Nat. Commun..

[28] Boyle AP (2012). Annotation of functional variation in personal genomes using RegulomeDB. Genome Res..

[29] Kent WJ (2002). The human genome browser at UCSC. Genome Res..

[30] Wingender E, Dietze P, Karas H, Knüppel R (1996). TRANSFAC: a database on transcription factors and their DNA binding sites. Nucleic Acids Res..

[31] GTEx Consortium. The Genotype-Tissue Expression (GTEx) project. *Nat. Genet*. **45**, 580–585 (2013). 10.1038/ng.2653 PMC4010069 23715323

[32] Lieberman-Aiden E (2009). Comprehensive mapping of long-range interactions reveals folding principles of the human genome. Science.

[33] Li Y, Willer C, Sanna S, Abecasis G (2009). Genotype imputation. Annu. Rev. Genomics Hum. Genet..

[34] Howie B, Marchini J, Stephens M (2011). Genotype imputation with thousands of genomes. G3 (Bethesda).

[35] 1000 Genomes Project Consortium, *et al*. A global reference for human genetic variation. *Nature* **526**, 68–74 (2015). 10.1038/nature15393 PMC4750478 26432245

[36] Li Y (2014). CRISPR reveals a distal super-enhancer required for Sox2 expression in mouse embryonic stem cells. PLoS One.

[37] Li X (2012). Genome-wide association studies of asthma indicate opposite immunopathogenesis direction from autoimmune diseases. J. Allergy Clin. Immunol..

[38] Schedel M (2015). Polymorphisms related to *ORMDL3* are associated with asthma susceptibility, alterations in transcriptional regulation of *ORMDL3*, and changes in TH2 cytokine levels. J. Allergy Clin. Immunol..

[39] Yang CY (2014). IL-12/Th1 and IL-23/Th17 biliary microenvironment in primary biliary cholangitis: implications for therapy. Hepatology.

[40] Hitomi Y (2015). Human primary biliary cholangitis-susceptible allele of rs4979462 enhances TNFSF15 expression by binding NF-1. Hum. Genet..

[41] Delaneau O, Zagury JF, Marchini J (2013). Improved whole-chromosome phasing for 50 disease and population genetic studies. Nat. Methods..

[42] Howie BN, Donnelly P, Marchini J (2009). A flexible and accurate genotype imputation method for the next generation of genome-wide association studies. PLoS Genet..

