# Supplementary material for: Identification of the functional variant driving ORMDL3 and GSDMB expression in human chromosome 17q12-21 in primary biliary cholangitis
Source: Sci Rep. 2017 Jun 6;7:2904. doi: 10.1038/s41598-017-03067-3 (PMC5460198; doi:10.1038/s41598-017-03067-3)

Identification of the functional variant driving *ORMDL3* and *GSDMB* expression in human chromosome 17q12-21 in primary biliary cholangitis

Yuki Hitomi[1,†,*], Kaname Kojima[2,3,†], Minae Kawashima[1,4], Yosuke Kawai[2,3], Nao Nishida[1,5], Yoshihiro Aiba[6], Michio Yasunami[7], Masao Nagasaki[2,3,8], Minoru Nakamura[6,9,10], Katsushi Tokunaga[1]

[1]Department of Human Genetics, Graduate School of Medicine, the University of Tokyo, Tokyo, Japan.

[2]Department of Integrative Genomics, Tohoku Medical Megabank Organization, Tohoku University, Sendai, Japan.

[3]Graduate School of Medicine, Tohoku University, Sendai, Japan.

[4]Japan Science and Technology Agency (JST), Kawaguchi, Japan.

[5]The Research Center for Hepatitis and Immunology, National Center for Global Health and Medicine, Ichikawa, Japan.

[6]Clinical Research Center, National Hospital Organization, Nagasaki Medical Center, Omura, Japan.

[7]Department of Clinical Medicine, Institute of Tropical Medicine, Nagasaki University, Nagasaki, Japan.

[8]Graduate School of Information Sciences, Tohoku University, Sendai, Japan.

[9]Department of Hepatology, Nagasaki University Graduate School of Biomedical Sciences, Omura, Japan.

[10]Headquarters of PBC Research in NHOSLJ, Clinical Research Center, National Hospital Organization Nagasaki Medical Center, Omura, Japan.

[*]Corresponding author

Yuki Hitomi, Ph.D

e-mail: yhitomi-tky@umin.ac.jp

[†]These authors contributed equally to this work

Supplementary Table 1. Oligo-nucleotide probes for the EMSA

**SNP**	Allele	Sequence (5' -> 3')
rs9303277	A	TTTTAACACAGCCCTATTTTAATTCATCGCA
	G	TTTTAACACAGCCCTGTTTTAATTCATCGCA
rs113897057	TAGAA	GTTCTTGATAGAGAATAGAAGATATGATCAATCAC
	del	GTTCTTGATAGAGAAGATATGATCAATCAC
rs2313430	C	TGAGTGAGATCTTTTCTTCATGTTCTTTTCG
	T	TGAGTGAGATCTTTTTTTCATGTTCTTTTCG
rs12946510	A	ATTTGCTGTGGTTTTATTTTTAACTCTGTCT
	G	ATTTGCTGTGGTTTTGTTTTTAACTCTGTCT

Supplementary Table 2. Primers for production of Luciferase assay constructs.

**SNP**	Name	Sequence (5' -> 3')
rs12946510	rs12946510-F1	GAGCTCTTTCCACTGCAGCCCTTTC
	rs12946510-R1	GGTACCGGGATCAGACAGTTGTTTGC
	rs12946510-F2	GGTACCTTTCCACTGCAGCCCTTTC
	rs12946510-R2	GAGCTCGGGATCAGACAGTTGTTTGC

Supplementary Figure 1 | LD (Linkage disequilibrium) structure around chr.17q12-21. Stronger colors indicate higher scores of D’ (**A**) and r^2 (**B**). A strong LD block in chr.17q12-21 included *ORMDL3*, *GSDMB*, *ZPBP7*, and *IKZF3*.

Supplementary Figure 2 | Result of a high-density association mapping in chromosome 17. High-density association mapping was based on SNP imputation analysis using 1KJPN and genotype data from our previous GWAS (PBC patients: n = 1,389; healthy controls: n = 1,508). Each dot shows the p-value of the SNPs. Red dots show experimentally genotyped SNPs by GWAS.

Supplementary Figure 3 | Luciferase reporter assay of rs12946510 using HuCCT1. The transcription enhancing activities of these plasmid constructs were measured by assay of the luciferase (luc) activity of transfected HuCCT1 24 hours after transfection. Cells transfected with the PBC-susceptible allele (T allele) of rs12946510 showed reduced luciferase activities compared to those transfected with the major allele (C allele). Three independent experiments were performed in each assay. Values of relative luciferase activity are shown as means ± SD. *P < 0.001 (Student’s t test)

Supplementary Figure 4 | | Luciferase reporter assay of rs12946510 using constructs that included the predicted enhancer region in an opposite orientation. The transcription enhancing activities of these plasmid constructs were measured by assay of the luciferase (luc) activity of transfected Jurkat, HepG2, and HuCCT1 cells, 24 hours after transfection. Cells transfected with the PBC-susceptible allele (T-allele) of rs12946510 showed reduced luciferase activities compared to those transfected with the major allele (C-allele). Three independent experiments were performed for each assay. Values of relative luciferase activity are shown as means ± SD. *P < 0.001 (Student's t test)

Supplementary Figure 5 | Differences were not observed in the endogenous expression levels of *IKZF3*, *GRB7*, and *GSDMA* in whole-blood among rs12946510 genotypes. Endogenous expression level of *IKZF3* (**A**), *GRB7* (**B**), and *GSDMA* (**C**) in whole-blood. There were no differences in the endogenous expression levels of these genes among rs12946510 genotypes. The statistical significance level after multiple comparison compensation by Bonferroni correction was P = 0.0045. These data were extracted from the GTEx portal database.

Supplementary Figure 6 | Differences were not observed in the endogenous expression levels of *IKZF3*, *ZPBP2*, *GRB7*, and *GSDMA* in the spleen among rs12946510 genotypes. The endogenous expression level of *IKZF3* (**A**), *ZPBP2* (**B**), *GRB7* (**C**), and *GSDMA* (**D**) in the spleen. There were no differences in the endogenous expression levels of these genes among rs12946510 genotypes. The statistical significance level after multiple comparison compensation by Bonferroni correction was *P* = 0.0045. These data were extracted from the GTEx portal database.

Supplementary Figure 7 | Chromatin interaction between rs12946510 and the upstream sequence of *ORMDL3* and *GSDMB*. Chromatin interaction between the 5 kb window that contains rs12946510 and the upstream sequence of *ORMDL3* and *GSDMB* could be detected from Hi-C database analysis (http://promoter.bx.psu.edu/hi-c/index.html). The color scale indicates the strength of the interaction. The blue circle indicates the interaction between rs12946510 and the upstream sequence of *ORMDL3* and *GSDMB.*

Supplementary Figure 1

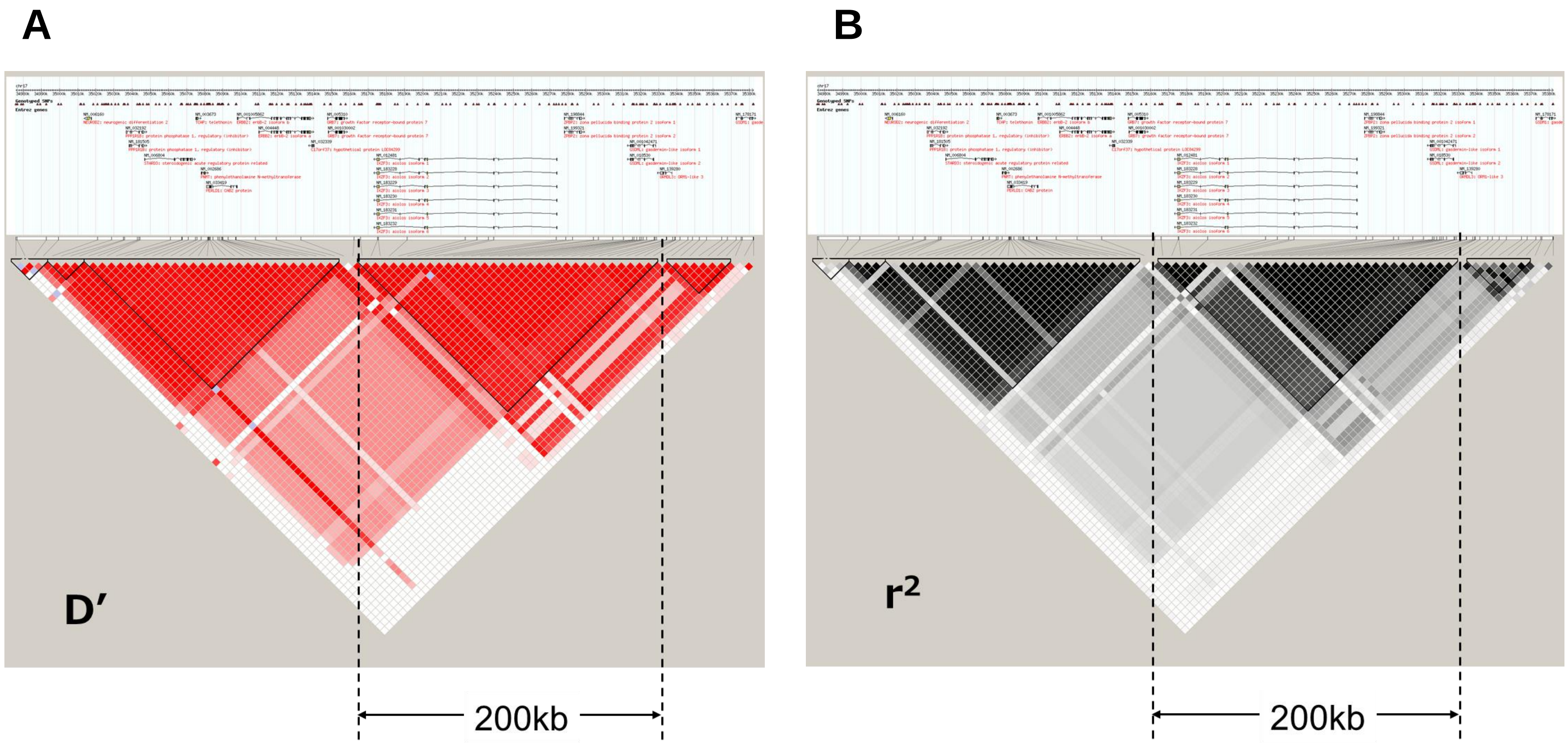

Supplementary Figure 2

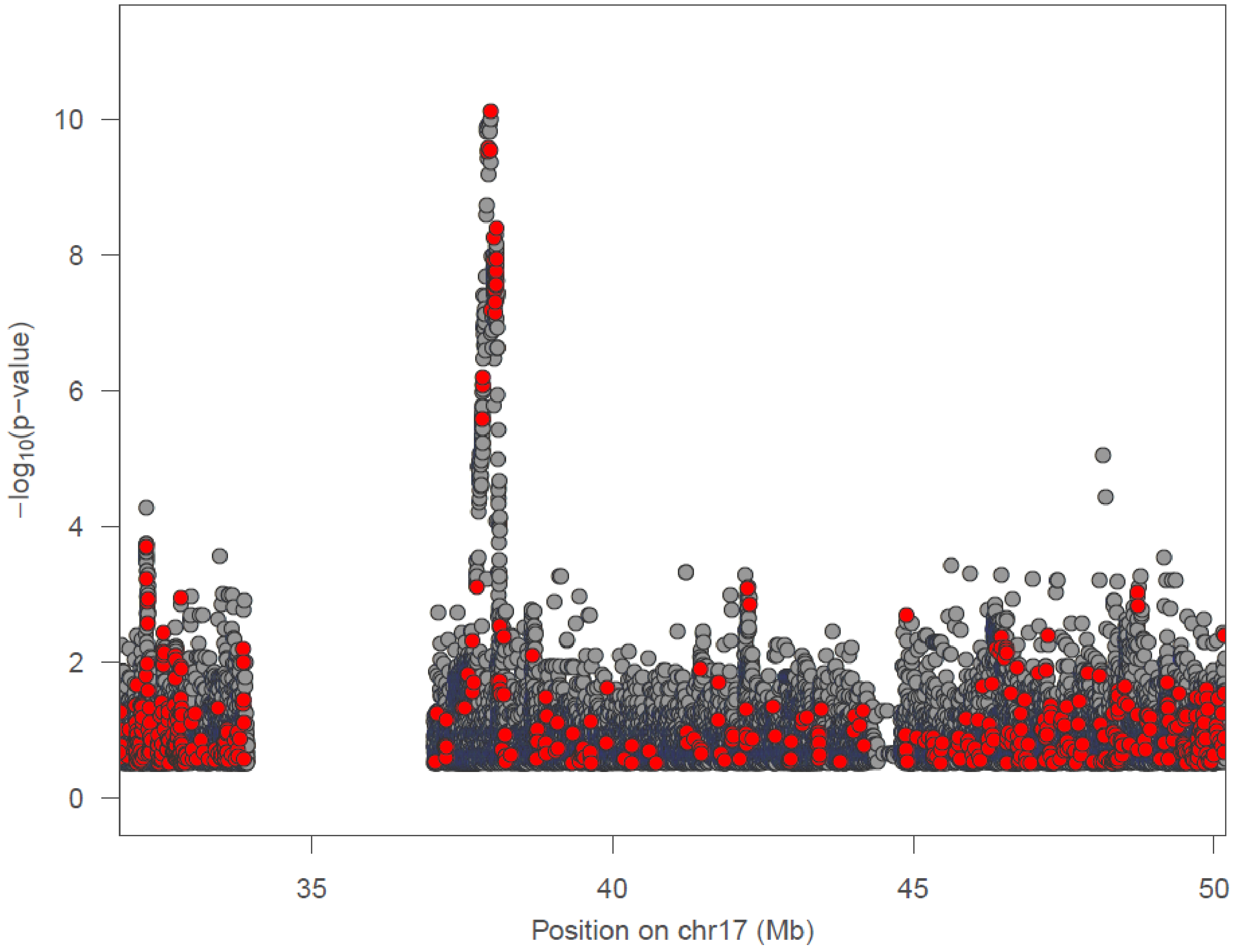

Supplementary Figure 3

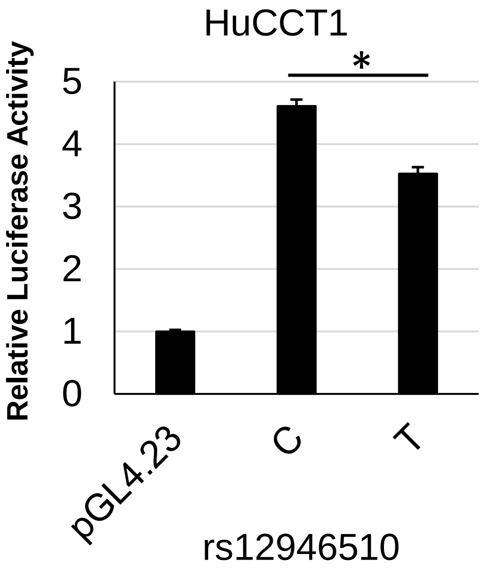

Supplementary Figure 4

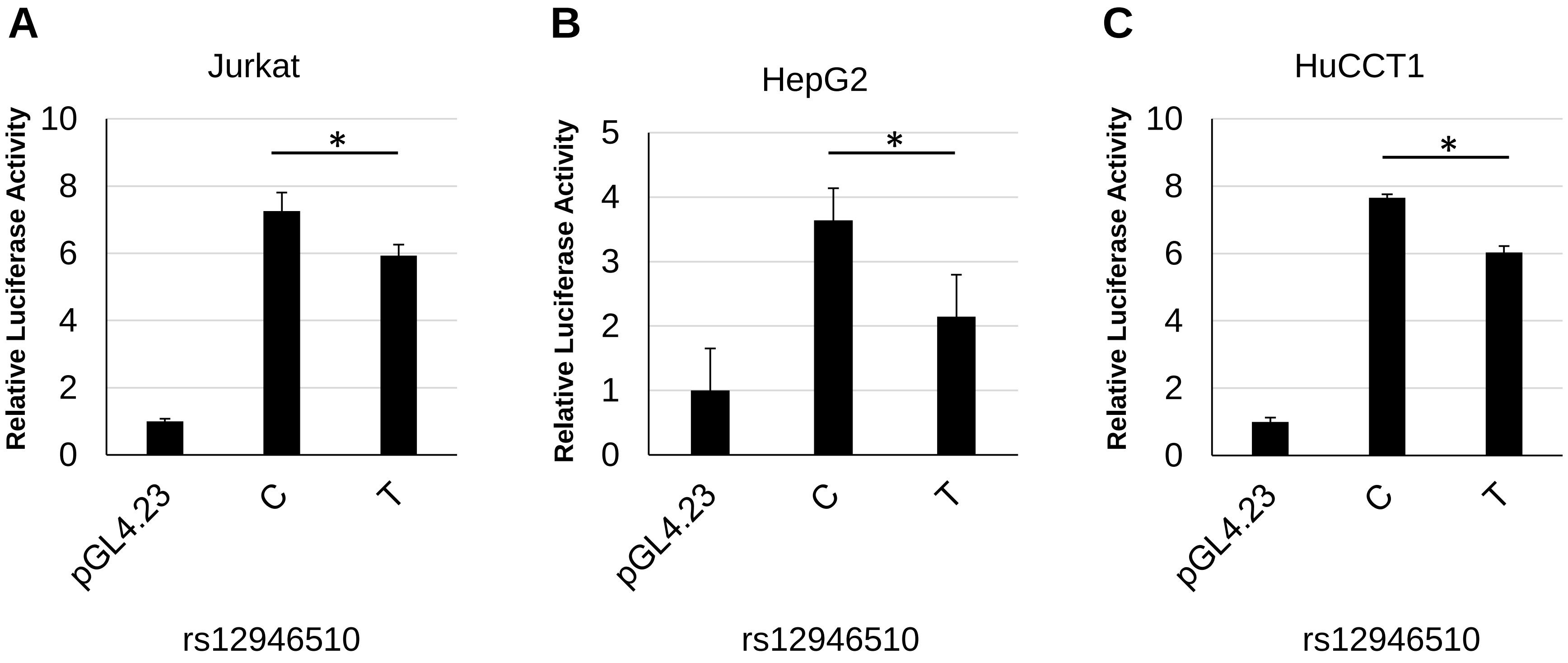

Supplementary Figure 5

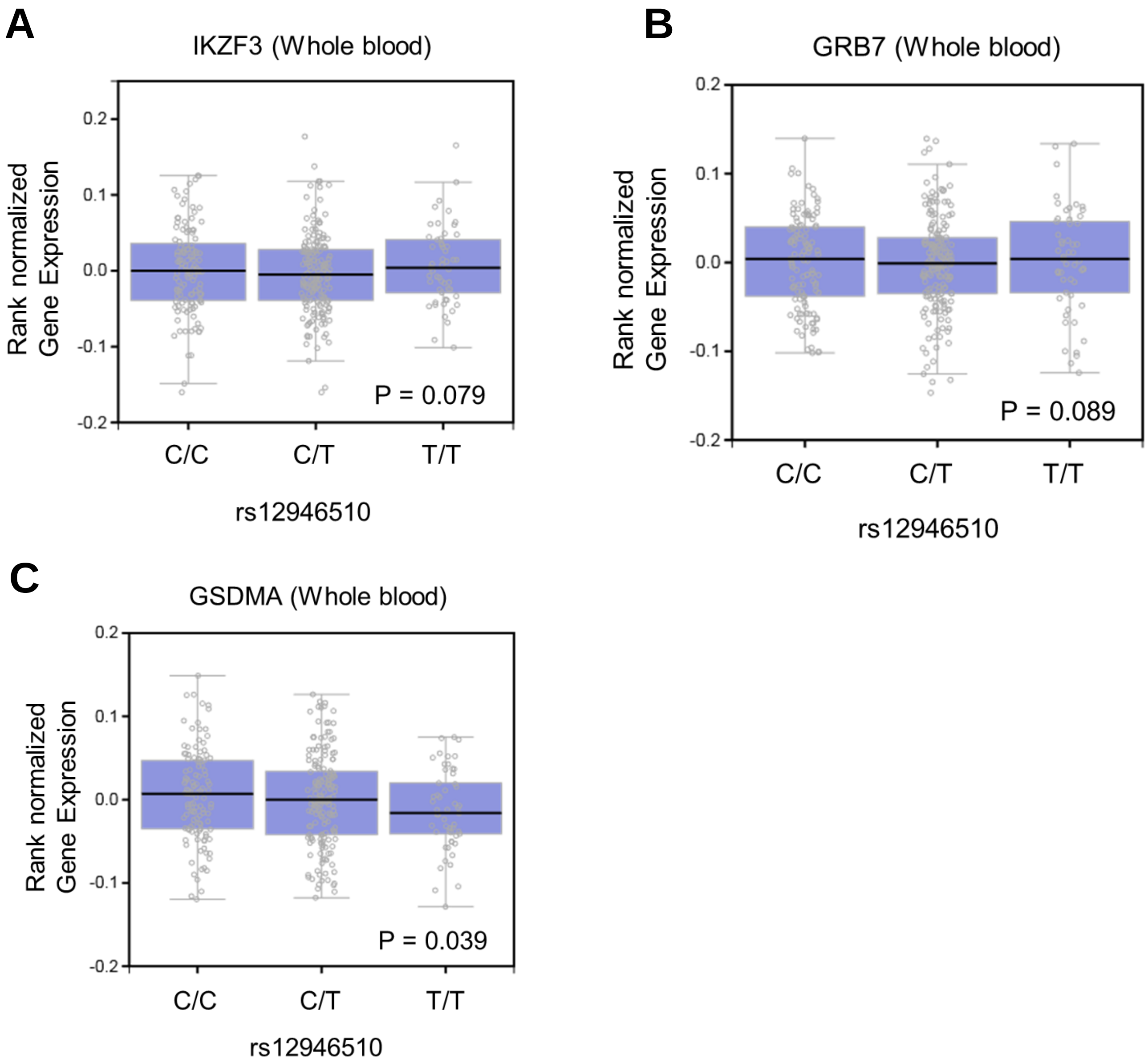

Supplementary Figure 6

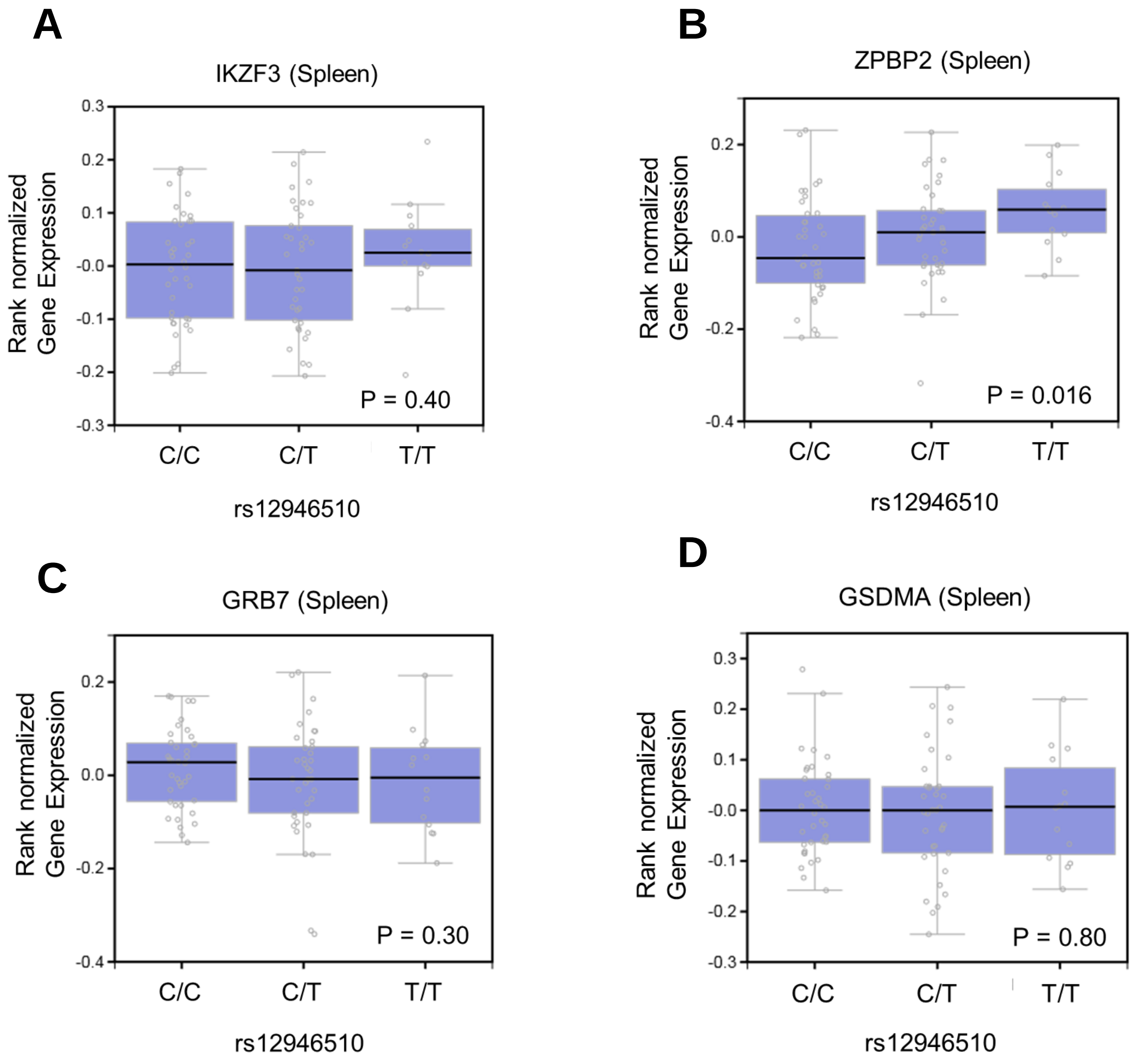

Supplementary Figure 7

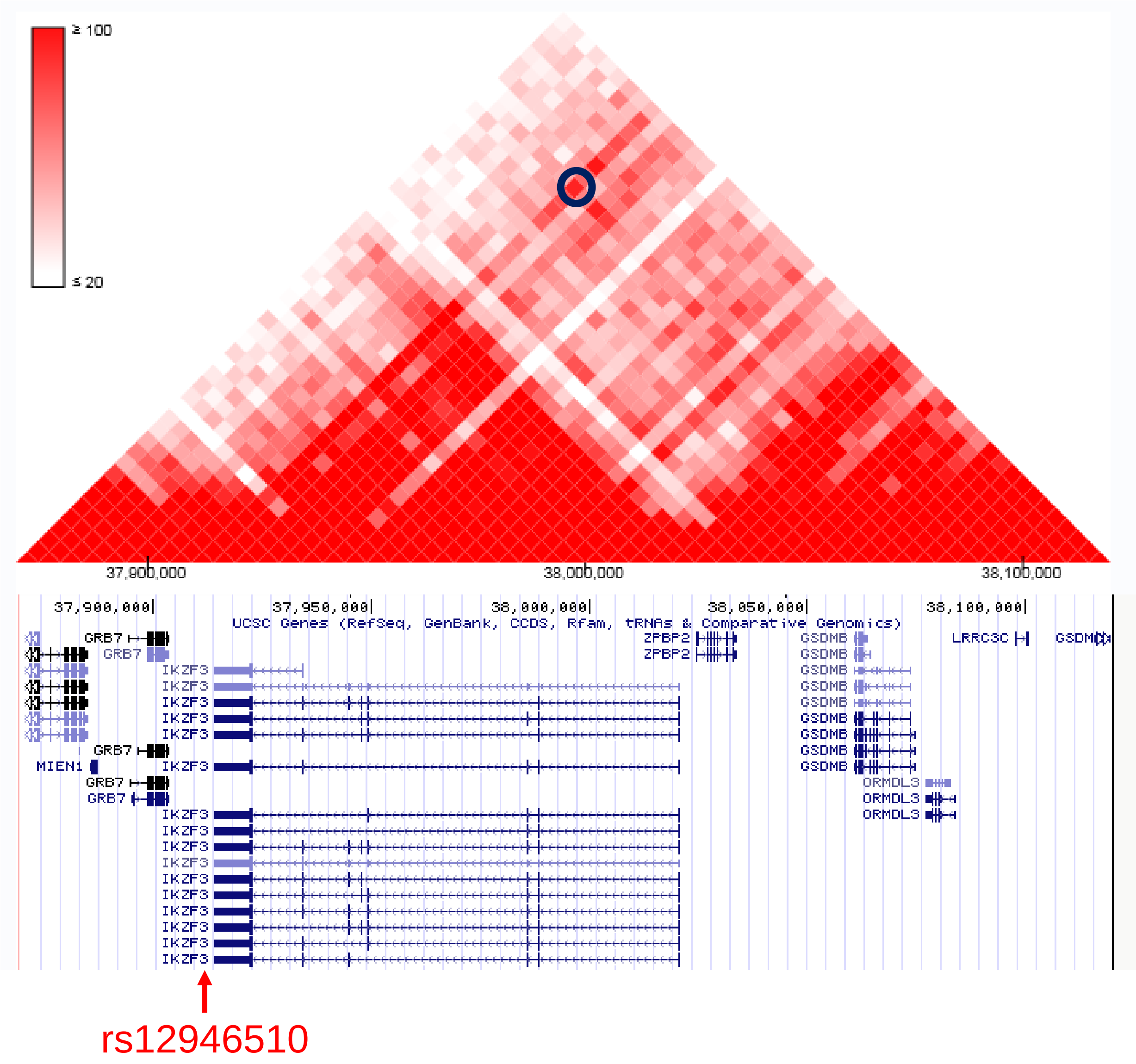

Supplement: Supplementary file 1 — Supplementary data [file 41598_2017_3067_MOESM1_ESM.pdf]
